# Congenital heart disease in men of reproductive age: Nationwide cohort studies of prevalence, male fertility, and birth outcomes in offspring

**DOI:** 10.1016/j.ijcchd.2025.100637

**Published:** 2025-11-06

**Authors:** Marit Sandberg, Nina Øyen, Tatiana Fomina, Ferenc Macsali, Gottfried Greve, Elisabeth Leirgul

**Affiliations:** a Department of Obstetrics and Gynecology, Haukeland University Hospital, Bergen, Norway; b Department of Global Public Health and Primary Care, University of Bergen, Norway; c Department of Medical Genetics, Haukeland University Hospital, Bergen, Norway; d Norwegian Institute of Public Health, Norway; e Department of Clinical Science, University of Bergen, Norway; f Department of Heart Disease, Haukeland University Hospital, Bergen, Norway

**Keywords:** Congenital heart disease, Men, Prevalence, Male fertility, Adverse birth outcomes, Epidemiology, Cohort study

## Abstract

**Background:**

The potential for men with congenital heart disease (CHD) to father children and the impact of paternal CHD on offspring birth outcomes are not well understood.

**Methods:**

Using Norwegian nationwide registries from 1994 to 2014, we identified two study populations: 1 829 877 men aged 18–50 years and 1 207 410 newborns. First, we reported the time trends of CHD in men and paternal CHD in newborns. Second, we compared the rate of becoming fathers in men with and without CHD. Third, we compared birth outcomes in offspring with and without paternal CHD.

**Results:**

Between 1994 and 2014, the prevalence of CHD in men of reproductive age increased from 22.7 to 32.2 per 10 000, while the prevalence of paternal CHD in newborns increased from 21.6 to 26.9 per 10 000. Men with mild CHD had a similar rate of becoming fathers compared to men without CHD (rate ratio 0.97, 95 % confidence interval 0.90–1.05), but men with moderate/severe CHD had a lower rate (rate ratio 0.78, 95 % confidence interval 0.70–87). Newborns with paternal CHD had no increased risk of preterm birth or being small for gestational age, compared to newborns without paternal CHD.

**Conclusion:**

The prevalence of CHD in men and paternal CHD in newborns was increasing. Moderate/severe CHD in men was associated with a lower rate of becoming fathers compared to the general male population. Paternal CHD was not associated with an increased risk of newborns being preterm or small for gestational age.

## Highlights

• The prevalence of congenital heart disease (CHD) in men increased from 1994 to 2014.
• Men with mild CHD had similar rates of becoming fathers as men without CHD.
• Men with moderate/severe CHD had lower birth rates and fewer children.
• The prevalence of paternal CHD in newborns increased from 1994 to 2014.
• Newborns with mild paternal CHD had no increased risk of adverse birth outcomes.

## Abbreviations

CHD: congenital heart disease
CI: confidence interval
MBRN: Medical Birth Registry of Norway
RR: risk ratio/rate ratio

## 1 Introduction

Congenital heart disease (CHD) represents a broad spectrum of conditions ranging from simple defects with minimal clinical impact to complex diseases requiring frequent hospitalizations and repeated surgery during childhood and adolescence. Advances in diagnostic methods and pediatric treatments have led to an increase in the prevalence of CHD among adults in recent decades. Consequently, the focus of healthcare has shifted from mere survival to enhancing the quality of life.

For many, establishing a family and having children is fundamental to life satisfaction. The prospect of becoming a parent depends on an interplay among biological, cultural, socioeconomic, and psychological factors — dimensions that are all integral to the concept of fertility. Many of these factors can be affected by the presence of a chronic health condition like CHD. For the offspring, parental genetic and lifestyle factors associated with CHD might contribute to adverse birth outcomes. Evidence on the impact of CHD on women's fertility is limited, but studies have demonstrated that newborns of mothers with CHD are at higher risk for preterm birth, low birth weight, and stillbirth. [[1], [2], [3], [4], [5], [6], [7]]. Corresponding knowledge regarding men remains scarce. The effects of CHD on men's fertility, as well as whether newborns of fathers with CHD face similar risks, remain largely unexplored.

In two nationwide cohorts involving men aged 18–50 years and their offspring, we aimed to address the following objectives: First, we reported the prevalence of CHD among men from 1994 to 2014 and reflected this on the prevalence of paternal CHD in newborns during the same period. Second, we assessed male fertility by comparing the rate of becoming fathers and the mean number of children in men with CHD and men without CHD. Finally, we assessed risks of adverse birth outcomes in newborns with and without paternal CHD.

## 2 Materials and methods

### 2.1 Study design and study population

**Fig. 1:**
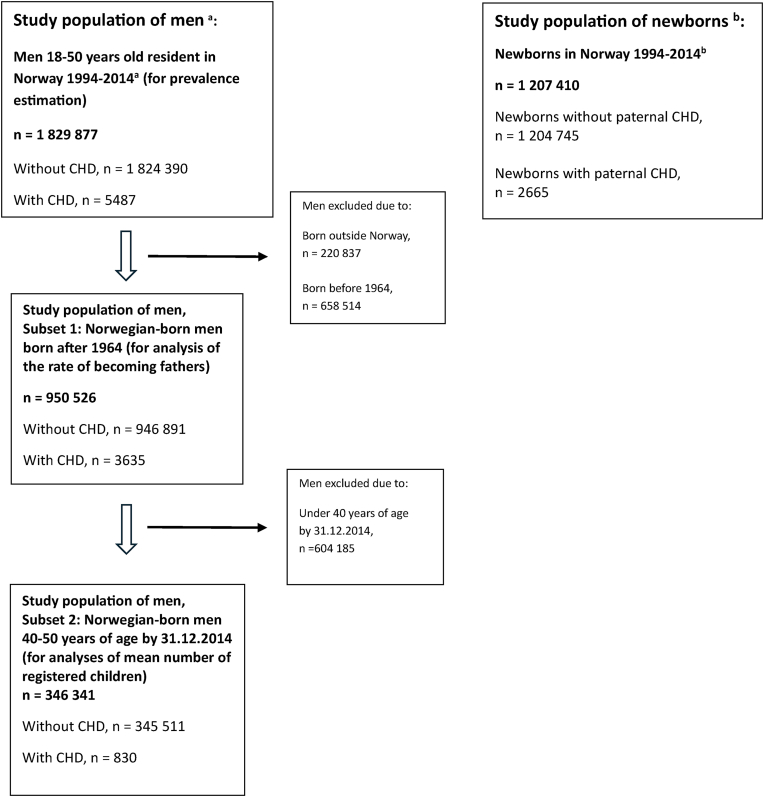
Study population of men and study population of newborns. Abbreviations: CHD, congenital heart disease ^a^ Men with Down syndrome were excluded from the analyses, as this condition is associated with both CHD and reduced reproductive potential. ^b^ Newborns by multifetal pregnancies and pregnancies with likely misclassified birthweight (birthweight by gestational age below or above four standard deviations from the mean) or gestational age >44 weeks were excluded from the analyses.

Using Norwegian national registries and a retrospective cohort design, we established two study populations (Fig. 1). First, we identified all male residents of reproductive age (18–50 years) from January 1, 1994, to December 31, 2014. We excluded men with Down syndrome, as this condition is associated with both CHD and reduced reproductive potential.

For the assessment of fertility, we used study population subsets of Norwegian-born men born after January 1, 1964.

Second, we identified all newborns from January 1, 1994, to December 31, 2014. Newborns by multifetal pregnancies and pregnancies with likely misclassified birthweight (birthweight by gestational age below or above four standard deviations from the mean) or gestational age >44 weeks were excluded from the analyses.

### 2.2 Data sources

The unique personal identification number assigned to all Norwegian residents enables linkage between the National Population Registry and other data sources. Statistics Norway contains demographic data. The Research in Hospital Database retrieved data on discharge diagnoses and procedures for individuals with cardiovascular diagnoses from somatic hospitals in Norway between 1994 and 2009 [8]. The Norwegian Patient Registry has been recording information on diagnoses and procedures from inpatient and outpatient treatment in the specialist healthcare service since 2008. The Cause of Death Registry retrieves the causes of death from death certificates. The Medical Birth Registry of Norway (MBRN) registers information on pregnancy, childbirth, and birth outcomes [9].

### 2.3 Identification and classification of CHD among men

Information on CHD was retrieved from the Research in Hospital Database, the Norwegian Patient Registry, the Cause of Death Registry, and the MBRN. We used codes for cardiac malformations in the International Classification of Diseases, Eighth, Ninth, and Tenth Revisions, as well as cardiac procedure codes from the NOMESCO Classification of Surgical Procedures and the Norwegian Classification of medical procedures, 3rd edition. Diagnoses of CHD were validated and prioritized based on the man's age at diagnosis and the source of the diagnosis, following a method described in a previous article (Table S1) [10]. To assign every man to a single cardiac phenotype, the recorded diagnoses and procedures from different classification systems were translated into cardiac phenotypes in a hierarchical approach using an algorithm developed for analyses of Norwegian and Danish register data (Table S1) [11]. Combined cardiac defects were coded according to the presumed first-appearing defect in embryologic cardiac development, usually representing the more complex defect.

The CHDs were divided into subgroups of mild and moderate/severe CHD (Table 1), broadly modelled on the European Society of Cardiology classification of congenital heart disease [12]. Due to the limited clinical information and surgical history available to differentiate between conditions, we combined moderate and severe CHD into a single group. Congenital heart diagnoses that could not be graded by severity were classified as other CHD (Table 1). Men with uncertain CHD status (a diagnosis of patent arterial duct only or an unspecified CHD recorded at birth but with no further registration of CHD) were excluded from the analyses, along with their offspring.

**Table 1:** Classification of mild CHD, moderate/severe CHD, and other CHD^a^.

Mild CHD	Atrial septal defect, ventricular septal defect, or selected valve defects (mitral or aortic insufficiency, pulmonary stenosis or insufficiency, or unspecified anomaly of the heart valves) unless these were combined with defects of higher priority in our classification system^a^.
**Moderate/severe CHD**	Heterotaxia with other heart defects, truncus arteriosus, transposition of the great arteries, tetralogy of Fallot, double outlet right ventricle, interrupted aortic arch, atrioventricular septal defect, total or partial anomalous pulmonary venous return, hypoplastic left heart syndrome, mitral stenosis/atresia, coarctation of aorta, supra valvular aorta stenosis, valvular aortic stenosis, hypoplastic right heart syndrome, tricuspid atresia, pulmonary atresia, valvular pulmonary atresia, Ebstein anomaly, and congenitally corrected transposition of the great arteries. In addition, procedures indicating Fontan circulation or mechanical valve replacement, or diagnoses indicating CHD with pulmonary hypertension were included in this group.
**Other CHD**	Unspecified congenital defects in the heart, unspecified congenital defects of great veins, and unspecified congenital defects of great arteries, unless these were combined with other defects of higher priority in our classification. In addition, coronary malformation and heterotaxy, unless these were combined with other defects of higher priority in our classification^a^^.^

Abbreviations: CHD, congenital heart disease.

a Further information on CHD is provided in Section 2.3, Identification and Classifications of CHD among men.

### 2.4 Outcome

**Study population of men**: Becoming a father was defined as being registered as the father in a live birth or stillbirth after the 22nd gestational week in MBRN. To analyze the mean number of children per man, all registered childbirths up to 2014 were recorded, and the paternal age at the first registered childbirth was calculated.

**Study population of newborns**: Birth outcome variables were retrieved from the MBRN. Gestational age was used to identify preterm birth (<37 weeks of gestation). Small for gestational age was defined as birthweight <10th percentile according to gestational age and fetal sex on national charts [13]. Stillbirth was death before delivery, and neonatal death was defined as death within 28 days after birth. Cesarean delivery included planned and emergency procedures. Apgar is a neonatal vitality assessment where a score <7 at 5 min is associated with an increased risk of neonatal mortality and morbidity [14]. In this study, such scores are defined as low. Neonatal intensive care unit admission implies a need for pediatric observation or treatment.

### 2.5 Covariates

**Study population of men**: We retrieved information on men's birth year, country of origin, marital/cohabitant status by December 31, 2014, and educational level by December 31, 2014 from Statistics Norway.

Men's birth year was considered a confounder in the association between CHD and the rate of becoming fathers, as the mean age of men with CHD in our cohort was lower than the reference population, and a younger age decreases the prospects of having fathered children.

Education and marital/cohabitant status were considered intermediate factors of the associations between CHD in men and birth rates. Still, we performed sensitivity analyses of the association between CHD and the rate ratio of becoming fathers with restrictions to men registered as married/cohabitant by December 31, 2014.

**Study population of newborns**: We retrieved information on the delivery year, fathers’ age, and parity (firstborn/later-born) from the MBRN.

We considered delivery year and parity as confounders in the association between paternal CHD and birth outcomes. Obstetric practices have changed over time, and the prevalence of paternal CHD increased during the study period. More newborns with paternal CHD were firstborns, and parity has been associated with birth outcomes.

### 2.6 Statistical analyses

**Study population of men:** The prevalence of CHD in men was estimated per 10 000 men of age 18–50 years from 1994 to 2014. The average annual percent change (AAPC) was estimated by Joinpoint regression [15] with a 95 % confidence interval (CI).

The rate of becoming fathers was estimated by the number of men with at least one registered childbirth during 1994–2014, divided by the total person-years of men. All men were followed from the start of the study period, January 1, 1994, or age 18 years, until the first registered childbirth, age 50 years, death, or the end of the study period, December 31, 2014, whichever came first. The association of CHD in men and becoming fathers was estimated by rate ratios (RR) with 95 % CI, comparing the rate of becoming fathers in men with CHD vs. in men without CHD using Poisson regression adjusted for men's year of birth.

The mean number of registered childbirths and the mean age at first childbirth were compared between men with and without CHD using Student's t-tests, and the proportions who had become fathers in different groups were compared using chi-square tests.

**Study population of newborns:** The prevalence of paternal CHD in newborns was estimated per 10 000 newborns born from 1994 to 2014. The average annual percentage change (AAPC) was estimated by Joinpoint regression [15] with a 95 % CI.

The proportions of preterm birth, small for gestational age, low Apgar score, neonatal intensive care unit admission, cesarean delivery, and stillbirth or neonatal death were calculated. The associations between paternal CHD and adverse birth outcomes were estimated using risk ratios (RR) with 95 % CI, comparing newborns with paternal CHD to newborns without paternal CHD, and were assessed using log-binomial regression adjusted for delivery year and parity.

The data linkage was performed with SAS version 9.4. Analyses were performed with STATA version 17 and the National Cancer Institute's Joinpoint Regression Program version 4.1.9.0.

## 3 Results

**Study population of men**: The study population of men 18–50 years of age between 1994 and 2014 consisted of 1 829 877 men after excluding individuals with Down syndrome (n = 370) (Fig. 1). In this population, 5487 men were registered with CHD (30 per 10 000); 3204 with mild CHD (18 per 10 000), 1811 with moderate/severe CHD (10 per 10 000) and 472 with other CHD (3 per 10 000).

**Fig. 2:**
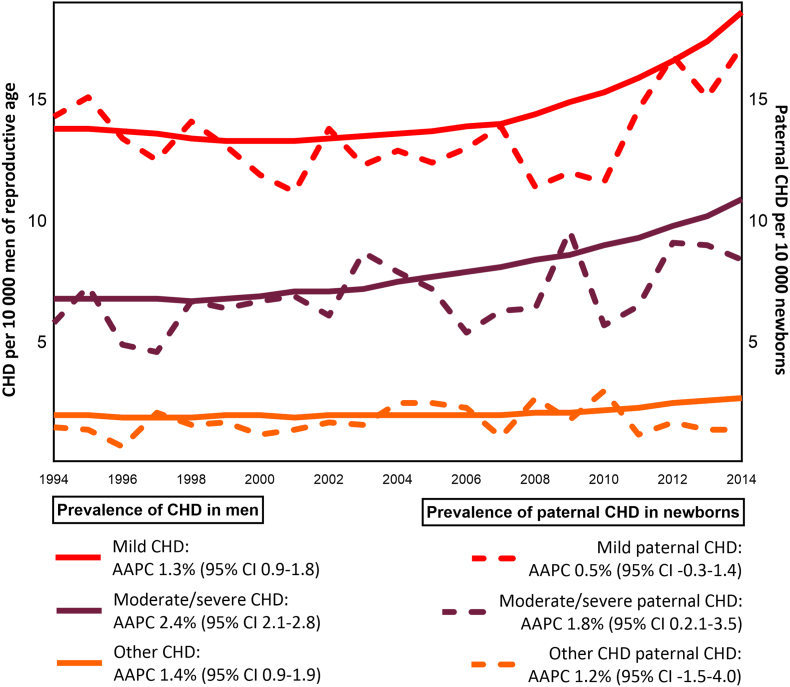
Prevalence of mild CHD, moderate/severe CHD, and other CHD per 10 000 men aged 18–50 years (left y-axis), and prevalence of mild paternal CHD, moderate/severe paternal CHD, and other paternal CHD per 10 000 newborns (right y-axis), in Norway, 1994–2014. Abbreviations: AAPC, average annual percent change; CHD, congenital heart disease, CI, confidence interval.

The total prevalence of CHD in men increased from 22.7 to 32.2 per 10 000 between 1994 and 2014. Mild CHD increased from 13.8 to 18.6 per 10 000 (AAPC 1.3 %, 95 % CI 0.9–1.8), moderate/severe CHD from 6.8 to 10.9 per 10 000 (AAPC 2.4 %, 95 % CI 2.1–2.8) other CHD from 2.0 to 2.7 per 10 000 (AAPC 1.4 %, 95 % CI 0.9–1.9) (Fig. 2).

In the subset of men born in Norway from 1964 onwards, we found a higher proportion of men with CHD who were born later in the study period, whereas men without CHD were born more evenly distributed across the study period (Table 2). Men with CHD had lower education levels, and 26–33 % were registered as married/cohabitant cohabiting by the end of the study period, compared with 47 % in the reference population.

**Table 2:** Distribution of covariates in 950 526 Norwegian-born men aged 18–50 years^a^, with mild, moderate/severe, or other CHD, and without CHD, in Norway 1994–2014.

		Men without CHD n = 946 891	Men with mild CHD n = 2032	Men with moderate/severe CHD n = 1288	Men with other CHD n = 315
**Year of birth n (%)**	1964–1974	345 511 (36.5)	462 (22.7)	292 (22.7)	76 (24.1)
	1975–1985	279 786 (29.6)	452 (22.2)	303 (23.5)	88 (27.9)
	1986–1996	321 594 (34.0)	1118 (55.0)	693 (53.8)	151 (47.9)
**Education by December 31, 2014**	<10 years	233 776 (24.7)	708 (34.8)	471 (36.6)	113 (35.9)
	11–13 years	425 223 (44.9)	789 (38.8)	429 (33.3)	117 (37.1)
	>14 years	273 776 (28.9)	468 (23.0)	249 (19.3)	53 (16.8)
	Missing	14 116 (1.5)	67 (3.3)	139 (10.8)	32 (10.2)
**Marital status by December 31, 2014**	Married/cohabitant	445 184 (47.0)	660 (32.5)	368 (26.2)	87 (27.6)
	Single/divorced/other	501707 (53.0)	1372 (67.5)	950 (73.8)	228 (72.4)

Abbreviations: CHD, congenital heart disease.

a Men born 01.01.1964–31.12.1996.

**Fig. 3:**
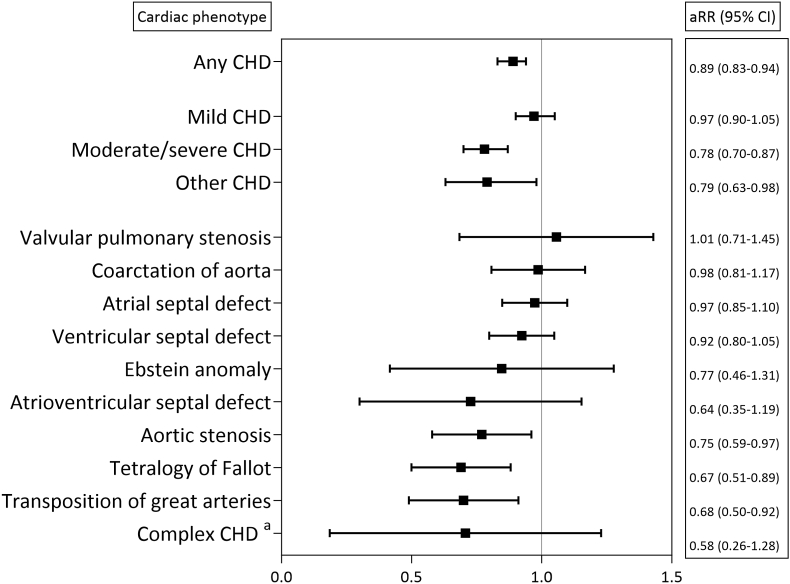
Rate ratio of becoming fathers in men with CHD compared to men without CHD adjusted for men's year of birth in 950 526 men at age 18–50 years in Norway, 1994–2014. ^a^Complex CHD consists of double outlet right ventricle, double outlet left ventricle, double inlet chamber, hypoplastic right heart syndrome, hypoplastic left heart syndrome, Fontan circulation, and mechanical valve replacements. Abbreviations: CHD, congenital heart disease; aRR, adjusted rate ratio; CI, confidence interval.

The rate ratios of becoming fathers between men with different CHD subgroups and men without CHD are presented in Fig. 3. Compared to men without CHD, men with mild CHD had similar rates of becoming fathers (aRR 0.97, 95 % CI 0.90–1.05), while men with moderate/severe CHD or other CHD had lower rates (aRR 0.78, 95 % CI 0.70–0.87 and aRR 0.79, 95 % CI 0.63–0.98). Restricting the analyses to men registered as married/cohabitant by the end of the study period, the lower rates of becoming fathers in men with moderate/severe and other CHD lost statistical significance (aRR 0.90, 95 % CI 0.80–1.01 and aRR 0.94, 95 % CI 0.74–1.19) (Table S2).

Men with and without CHD were of similar age when fathering their first child. The mean age was 28.9 years in men without CHD, 28.6 years in men with mild and moderate/severe CHD, and 28.9 years in men with other CHD (Table S3).

Table 3 demonstrates fatherhood in men who were 40–50 years of age as of 31.12.14. The proportions of men who were childless were comparable between men without CHD and men with mild CHD (22.4 % and 23.4 %, respectively), but higher among men with moderate/severe CHD and other CHD (34.9 % and 28.9 %, respectively). Similarly, the mean number of children was comparable between men without CHD (1.75, 95 % CI 1.75–1.75) and men with mild CHD (1.76, 95 % CI 1.64–1.88), but lower in men with moderate/severe CHD (1.41, 95 % CI 1.27–1.56) or other CHD (1.47, 95 % CI 1.18–1.74).

**Table 3:** Fatherhood in men with mild, moderate/severe or other CHD, compared to men without CHD, in 346 341 men aged 40–50 years in Norway^a^.

	Men without CHD n = 345 511	Men with mild CHD n = 462		Men with moderate/severe CHD n = 292		Men with other CHD n = 76	
	n (%)	n (%)	p-value	n (%)	p-value	n (%)	p-value
**Have become fathers, n (%)**	268 275 (77.6)	354 (76.6)	0.602	190 (65.1)	<0.001	54 (71.1)	0.168
**Remained childless, n (%)**	77 236 (22.4)	108 (23.4)		102 (34.9)		22 (28.9)	

**Mean number of children, (95 % CI)**	1.75 (1.75–1.75)	1.76 (1.64–1.88)	0.860	1.41 (1.27–1.56)	<0.001	1.47 (1.18–1.74)	0.0432
**Mean number of children in those having at least one child, (95 % CI)**	2.25 (2.25–2.25)	2.30 (2.19–2.40)	0.366	2.17 (2.05–2.30)	0.245	2.05 (1.81–2.30)	0.115


Abbreviations: CHD, congenital heart disease.

a As of December 31, 2014.

**Study population of newborns**: The study population of newborns consisted of 1 207 410 newborns after excluding newborns by multiple births (n = 20 240), and those with likely misclassification of gestational age or birthweight (n = 32 281) (Fig. 1). In this population, 2665 newborns were registered with paternal CHD (22 per 10 000 births); 1559 with mild paternal CHD (13 per 10 000), 800 with moderate/severe paternal CHD (7 per 10 000) and 195 with other paternal CHD (2 per 10 000).

The total prevalence of paternal CHD in newborns increased from 21.6 to 26.9 per 10 000 between 1994 and 2014. Moderate/severe paternal CHD increased from 6.8 to 10.9 per 10 000 (AAPC 1.8 %, 95 % CI 0.2–3.5). In contrast, the change in prevalence of mild paternal CHD (from 14.3 to 17.2 per 10 000) and other paternal CHD (from 1.5 to 1.6 per 10 000) was not statistically significant (Fig. 2).

A larger proportion of newborns with paternal CHD were delivered in the later years of the study, whereas births without paternal CHD were evenly distributed throughout the study period (Table S4). While 41 % of newborns without paternal CHD were firstborns, 42–44 % were firstborns among newborns with paternal CHD.

Adverse birth outcomes in offspring with and without paternal CHD are presented in Table 4. We found no associations between paternal CHD and each of the following outcomes: preterm birth, small for gestational age, low Apgar score, admission to neonatal intensive care units, or cesarean delivery. There were thirteen cases of stillbirth or neonatal death in newborns with mild or moderate/severe paternal CHD, indicating no increased risk compared to newborns without paternal CHD. In contrast, among newborns with other paternal CHD, we observed a threefold increased risk of stillbirth or neonatal death (aRR 3.11, 95 % CI 1.01–9.56). However, this estimate was based on fewer than five cases, resulting in a wide confidence interval.

**Table 4:** Risk ratios of adverse birth outcomes in newborns with mild, moderate/severe and other paternal CHD compared to newborns without paternal CHD, adjusted for year of delivery and parity of childbirth, among 1 207 410 newborns in Norway 1994–2014.

	Newborns without paternal CHD n = 1 152 335	Newborns with paternal CHD					
		Mild CHD n = 1559		Moderate/severe CHD n = 800		Other CHD n = 195	
	n (%)	n (%)	aRR^b^ (95 % CI)	n (%)	aRR^b^ (95 % CI)	n (%)	aRR^b^ (95 % CI)
**Preterm birth**	60935 (5.3)	72 (4.6)	0.88 (0.70–1.10)	45 (5.6)	1.06 (0.80–1.41)	8 (4.1)	0.77 (0.39–1.52)
**Small for gestational age**	119 159 (10.2)	165 (10.4)	1.02 (0.89–1.18)	89 (10.9)	1.02 (0.83–1.25)	21 (10.5)	0.96 (0.62–1.48)
**Stillbirth or neonatal death**	5711 (0.5)	8 (0.5)	1.04 (0.52–2.07)	5 (0.6)	1.27 (0.53–3.05)	<5 (1.5)	3.11 (1.01–9.56)
**Cesarean delivery**	163 457 (14.2)	224 (14.4)	1.01 (0.89–1.14)	97 (12.1)	0.85 (0.70–1.02)	30 (15.4)	1.08 (0.78–1.50)
**Apgar score < 7 after 5 min**^a^	10 892 (1.0)	18 (1.21)	1.22 (0.77–1.93)	10 (1.32)	1.31 (0.71–2.43)	<5 (0.53)	0.53 (0.08–3.74)
**NICU admission**^a^^b^	43 621 (5.1)	70 (6.2)	1.21 (0.97–1.52)	52 (8.5)	1.66 (1.28–2.16)	10 (6.8)	1.31 (0.73–2.39)

Abbreviations: aRR, adjusted risk ratio; CHD, congenital heart disease; CI, confidence interval; NICU, neonatal intensive care unit.

a Restricted to newborns born from the 37th gestational week.

b Restricted to newborns born after 1998 due to changed registration practices.

## 4 Discussion

In two nationwide cohorts, the prevalence of CHD in men and the prevalence of paternal CHD in newborns increased from 1994 to 2024. The rate of becoming fathers and the mean number of children were reduced in men with moderate/severe and other CHD, whereas results were similar in men with mild CHD compared to the reference population. Paternal CHD was not associated with adverse birth outcomes such as preterm birth, small for gestational age, low Apgar score, or cesarean delivery. The risks of neonatal intensive care unit admission and stillbirth or neonatal death were increased in paternal CHD subgroups.

Supporting our results, several international cohort studies have reported an increasing prevalence of CHD in adults [16,17]. Compared to our previous study of CHD in *women* of reproductive age [10], the prevalence of CHD in women was somewhat elevated compared to that of men. As some CHD diagnoses in women were detected solely through registrations due to pregnancy and childbirth, we could expect that the actual prevalence of CHD in men was slightly higher than what we present in this study. To our knowledge, the proportion of men with CHD who become fathers, and the resulting prevalence of paternal CHD in newborns, have not been previously reported.

Regarding fertility in men, our result of CHD in men being associated with a 0.9-fold decreased rate ratio of becoming fathers was supported by other studies. A Swedish cohort study comparing men with CHD to men without CHD found a 0.8-fold decreased incidence of becoming fathers among men aged 24–34 years [18]. A Danish cohort study found a lower total birth rate ratio of 0.9 in men aged 34 to 41 [19] while another Danish cohort study found a 0.8-fold lower incidence of becoming fathers in men born between 1970 and 2011 [20]. Our finding that the severity of CHD in men aligned with a reduced rate of fathering children was supported by the Danish study [20]. The Swedish study reported an increased proportion of childlessness with increasing CHD complexity [19].

We expected that the presence of CHD would have a weaker influence on men than on women on the ability and wish to become parents. In women with CHD, higher rates of menstrual disturbances might reduce the possibility of conceiving [21]. The substantial hemodynamic changes induced and necessitated in pregnancy pose risks for the woman, and several cohort studies have reported an elevated risk of fetal complications [[1], [2], [3], [4], [5]]. The recurrence of heart defects is higher with maternal CHD than with paternal CHD [20].

However, in our previous article on CHD in women, we found a comparable decreased rate of becoming mothers in women with CHD relative to women without CHD [10], as the decreased rate in men with CHD relative to men without CHD found in this study. Further, the mean number of registered childbirths was similarly reduced in men and women with moderate/severe CHD compared to men and women without CHD [10]. This finding was supported by the Danish study, which found a similarly decreased mean number of children in men and women with CHD compared to the reference population [19].

Given this information, it is reasonable to expect that men and women with CHD share underlying mechanisms influencing their fertility. Growing up and living with a chronic disease might affect many aspects of life. Elevated risks of neurocognitive and psychosocial impairments have been reported in individuals with CHD [[22], [23], [24]], potentially complicating the pursuit of sexual health, intimate relationships, and family life [24]. In this article, we report a lower proportion of marriage/cohabitation among men with CHD compared to unaffected men, mirroring our earlier finding in women with CHD [10]. Moreover, men with CHD who were married or cohabiting at the end of the study period had fatherhood rates comparable to those of men without CHD. Despite the acknowledged importance of relationships and sexuality to quality of life, these topics might often be unspoken in patient education and counseling of adolescent men with CHD. The decision not to establish relationships and have children may also reflect a personal choice—among both men and women—arising from concerns about future health and the ability to care for a child.

The null association between paternal CHD and most adverse birth outcomes in offspring in the present study was supported by the Swedish study, which reported no differences in preterm birth, small for gestational age, or cesarean delivery in children of men with and without CHD [18]. This encouraging information should be emphasized in counseling men with CHD and their partners who consider having children. The fact that CHD in women [[1], [2], [3], [4], [5], [6]] but not in men is associated with adverse birth outcomes might suggest that suboptimal intrauterine conditions, rather than a transmission of genes, are risk factors for adverse birth outcomes in women with CHD. Still, for rare outcomes such as stillbirth and neonatal death, confidence intervals were wide. Both the absence of increased risk in newborns with mild or moderate/severe CHD and the threefold increased risk observed in those with other paternal CHD should therefore be interpreted with caution.

Our study had some limitations. First, some diagnoses of mild CHD could have been missed if they were inpatient diagnoses made before 1994 or outpatient diagnoses before 2008. Still, as we detected CHD registered for more than two decades and used the unique personal identification number to link data from several national registries, we believe we have included most minor CHD. Second, there could be misclassified CHD. However, we evaluated and prioritized the diagnosis based on the men's age at the time of diagnosis and the department from which the diagnosis originated. Third, given Norway's universal healthcare system, our findings on fertility may not be generalizable to settings with more limited access to somatic and psychological follow-up for adolescents with CHD. Fourth, while CHD in men has been associated with a threefold increased risk of recurrence in offspring [20], this aspect could not be examined in our study, and we therefore cannot determine whether CHD in offspring affects birth outcomes in this group. Men registered as fathers in MBRN have not been verified as biological fathers, but a genetic study from Iceland, a society with similarities to Norway, reported a nonpaternity rate of only 1.5 % [25]. As we do not expect this rate to differ between men with and without CHD, any misclassification due to non-paternity would likely be non-differential. Still, if non-paternity rates were higher among men with CHD, genetic transmission of CHD would be reduced, potentially leading to more favorable birth outcomes among their registered offspring. Fifth, information on living areas was unavailable. Individuals requiring frequent hospital visits may prefer to live closer to major hospitals, which could represent a residual confounder in the analyses of birth outcomes in offspring. Finally, the limited diagnostic specificity of defects classified as other CHD poses challenges for the clinical interpretation of findings within this group.

The major strength of our study was the use of individual-level data in large and unselected national cohorts. The universal health care system, with virtually no private alternative for congenital heart surgery, minimizes bias due to selection. For birth outcomes, the validity of gestational age and birthweight in MBRN is strong [26].

## 5 Conclusion

The prevalence of CHD in men of reproductive age and the prevalence of paternal CHD in newborns increased from 1994 to 2014. Men with moderate/severe CHD had a reduced rate of becoming fathers and a lower mean number of children, while men with mild CHD had the same rate of becoming fathers and the same mean number of children compared to men without CHD. Paternal CHD was not associated with increased risks of newborns being preterm or small for gestational age. As family, sexuality, and fertility are considered important for life quality by many, addressing these fields in patient education and counseling is needed to empower young men with CHD.

## CRediT authorship contribution statement

**Marit Sandberg:** Writing – review & editing, Writing – original draft, Visualization, Methodology, Formal analysis, Conceptualization. **Nina Øyen:** Writing – review & editing, Supervision, Project administration, Methodology, Conceptualization. **Tatiana Fomina:** Writing – review & editing, Software, Methodology, Data curation. **Ferenc Macsali:** Writing – review & editing, Conceptualization. **Gottfried Greve:** Writing – review & editing, Conceptualization. **Elisabeth Leirgul:** Writing – review & editing, Supervision, Methodology, Conceptualization.

## Ethics statement

The Regional Ethics Committee of Western Norway approved the study on March 12, 2015 (REK 111545). The use of Norwegian national register data does not require consent from participants.

## Sources of funding

Financial support from the 10.13039/100002129Heart Foundation at the 10.13039/501100005036University of Bergen, the 10.13039/501100008568Norwegian Women's Public Health Association, and the Norwegian Association for Children with Congenital Heart Disease. The funders had no role in the study design, analysis, or preparation of the manuscript.

## Declaration of competing interest

The authors declare that they have no known competing financial interests or personal relationships that could have appeared to influence the work reported in this paper.
