# Supplementary material for: Congenital heart disease in men of reproductive age: Nationwide cohort studies of prevalence, male fertility, and birth outcomes in offspring
Source: Int J Cardiol Congenit Heart Dis. 2025 Nov 6;22:100637. doi: 10.1016/j.ijcchd.2025.100637 (PMC12664383; doi:10.1016/j.ijcchd.2025.100637)
Supplement: Multimedia component 1 [file mmc1.docx]

**Supplementary material:** Congenital heart disease in men of reproductive age: Nationwide cohort studies of prevalence, male fertility and birth outcomes in offspring.

**Table S1: Identification and classification of congenital heart disease ^a^.**

| CHD was ascertained from the Medical Birth Registry of Norway (1), the Research in Hospital database (2), the Norwegian Patient Registry (3), and the Cause of Death Registry (4) using cardiac defect codes in the International Classification of Diseases 8th, 9th, and 10th Revisions and cardiac procedure codes by the NOMESCO Classification of Surgical Procedures and the Norwegian Classification of Medical Procedures 3rd edition.  We recorded all CHD diagnoses given to men <18 years old. To avoid misclassifications, we only registered CHD diagnoses in men ≥18 years from pediatric, cardiology, thoracic surgery, or internal medicine departments. For men 30 years or older, we did not count diagnoses of congenital valve defects if an acquired valve defect was recorded first. Patent arterial duct or unspecified CHD recorded at birth only were not regarded as birth defects.    If a man was recorded with successive diagnoses of CHD, we prioritized the source of diagnosis as follows: first entry at university hospitals, first entry at regional/local hospitals, the Medical Birth Registry of Norway, the Cause of Death Registry, procedure codes from university hospitals, and procedure codes from regional/local hospitals.    Each man was assigned a single cardiac phenotype in a hierarchical approach using an algorithm developed for analyzing Norwegian and Danish register data (5). Combined defects were coded according to the embryological first–appearing condition, which usually represented the more complex defect.  The cardiac diagnoses were subsequently classified as mild and moderate/severe, broadly modelled on the European Society of Cardiology Guidelines for Adult Congenital Heart Disease classification (6). We merged moderate and severe CHD into one group due to a limited number of severe diagnoses and a lack of clinical information and surgical history to distinguish between moderate and severe CHD. One out of four men with moderate/severe CHD was likely to have severe CHD, according to the European Society of Cardiology classification (6). Additionally, some cardiac diagnoses were classified as other CHD. This group includes unspecified diagnoses of congenital defects involving the heart, great veins, and great arteries, as well as coronary malformations and heterotaxy, unless these were combined with other defects of higher priority in our classification system. |
| --- |
| 1. Irgens LM. The Medical Birth Registry of Norway. Epidemiological research and surveillance throughout 30 years. Acta Obstet Gynecol Scand. 2000;79(6):435-9.  2. Sulo G, Igland J, Vollset S, Nygård O, Øyen N, Tell G. Cardiovascular disease and diabetes mellitus in Norway during 1994–2009 CVDNOR – a nationwide research project. Norwegian journal of epidemiology. 2013;23:101-7.  3. Helsedata. Norwegian Patient Registry (NPR) [Internet]. Helsedata; [Available from: <https://helsedata.no/en/forvaltere/norwegian-directorate-of-health/norwegian-patient-registry-npr/>.  4. Helsedata. Norwegian Cause of Death Registry [Internet]. Helsedata; [Available from: <https://helsedata.no/en/forvaltere/norwegian-institute-of-public-health/norwegian-cause-of-death-registry/>.  5. Leirgul E, Fomina T, Brodwall K, Greve G, Holmstrøm H, Vollset SE, et al. Birth prevalence of congenital heart defects in Norway 1994-2009--a nationwide study. Am Heart J. 2014;168(6):956-64.  6. Baumgartner H, De Backer J, Babu-Narayan SV, Budts W, Chessa M, Diller GP, et al. 2020 ESC Guidelines for the management of adult congenital heart disease. Eur Heart J. 2021;42(6):563-645. |

Abbreviations: CHD, congenital heart disease

^a^ The identification and classification of CHD were also presented in *Sandberg M, Fomina T, Macsali F, Greve G, Øyen N, Leirgul E. Preeclampsia and neonatal outcomes in pregnancies with maternal congenital heart disease: A nationwide cohort study from Norway. Acta Obstet Gynecol Scand. 2024.*

**Table S2.** Rate ratio of becoming fathers in men with CHD compared to men without CHD adjusted for men’s year of birth with restrictions to men registered as married/cohabitant by 31.12.2024 in 446 299 men at age 18–50 years in Norway, 1994–2014.

|  | Rate ratio of becoming fathers | |
| --- | --- | --- |
|  | aRR | 95% CI |
| Men without heart disease  n = 445 184 | (ref) | (ref) |
| Men with mild CHD  n = 660 | 1.04 | 0.96–1.14 |
| Men with moderate/severe CHD  n = 368 | 0.90 | 0.80–1.01 |
| Men with other CHD  n = 87 | 0.94 | 0.74–1.19 |

Abbreviations: CHD, congenital heart disease; aRR, adjusted rate ratio; CI, confidence interval.

**Table S3.** Men’s mean age at first childbirth among 390 124 Norwegian-born men aged 18–50 years, 1994–2014 ^a^.

|  | Mean age | Difference (days) ^b^ | p-value |
| --- | --- | --- | --- |
| Men without CHD  n = 389 159 | 28.9 | (ref) | (ref) |
| Men with mild CHD  n = 560 | 28.6 | -51 | 0.458 |
| Men with  moderate/severe CHD  n = 324 | 28.6 | -62 | 0.458 |
| Men with other CHD  n = 81 | 28.9 | -21 | 0.898 |

Abbreviations: CHD, congenital heart disease.

^a^ Born 01.01.1964-31.12.1996

^b^ Difference (days) estimated by linear regression adjusted for man’s birth year.

**Table S4:** Covariates of newborns with mild, moderate/severe or other paternal CHD, and without paternal CHD, among 1 207 410 newborns in Norway, 1994–2014.

|  | | Newborns without paternal CHD  n = 1 152 335 | Newborns with paternal CHD | | |
| --- | --- | --- | --- | --- | --- |
|  |  |  | Mild CHD  n = 1559 | Moderate/severe CHD  n = 800 | Other CHD  n = 195 |
| **Delivery year**  **n (%)** | 1994-1996 | 156 232 (13.6) | 230 (14.8) | 93 (11.6) | 19 (9.7) |
|  | 1997-1999 | 158 761 (13.8) | 208 (13.3) | 95 (11.9) | 30 (15.4) |
|  | 2000-2002 | 162 300 (14.1) | 202 (13.0) | 107 (13.4) | 22 (11.3) |
|  | 2003-2005 | 162 182 (14.1) | 217 (13.9) | 127 (15.9) | 35 (18.0) |
|  | 2006-2008 | 169 491 (14.7) | 222 (12.2) | 102 (12.8) | 33 (16.9) |
|  | 2009-2011 | 174 779 (15.2) | 281 (18.0) | 128 (16.0) | 34 (11.3) |
|  | 2012-2014 | 168 590 (14.6) | 469 (19.3) | 148 (18.5) | 22 (21.3) |
| **Paternal age (years) at delivery**  **mean (SD)** | | 32.4 (6.1) | 32.4 (6.5) | 31.7 (6.3) | 31.7 (5.8) |
| **Paternal age (years) at delivery, categories**  **n (%)** | ≤19 | 7204 (0.6) | 16 (1.0) | 12 (1.5) | 2 (1.0) |
|  | 20-24 | 88 047 (7.6) | 147 (9.4) | 83 (10.4) | 19 (9.7) |
|  | 25-29 | 289 037 (25.1) | 380 (24.4) | 208 (26.0) | 51 (26.2) |
|  | 30-34 | 388 252 (33.7) | 493 (31.6) | 257 (32.1) | 67 (34.4) |
|  | 35-39 | 244 969 (21.3) | 324 (20.8) | 157 (19.6) | 38 (19.5) |
|  | 40-44 | 94 021 (8.2) | 132 (8.5) | 55 (6.9) | 14 (7.2) |
|  | ≥45 | 40 805 (3.5) | 67 (4.3) | 28 (3.5) | 4 (2.1) |
| **Parity of childbirth**  **n (%)** | Firstborn | 493 524 (41.0) | 682 (42.0) | 368 (44.0) | 88 (43.4) |
|  | Later-born | 711 221 (59.0) | 943 (58.0) | 469 (56.0) | 115 (56.7) |

Abbreviations: CHD, congenital heart disease; SD, standard deviation
